# Modulation of Bacterial Fitness and Virulence Through Antisense RNAs

**DOI:** 10.3389/fcimb.2020.596277

**Published:** 2021-02-11

**Authors:** Jess A. Millar, Rahul Raghavan

**Affiliations:** ^1^ Department of Epidemiology, University of Michigan, Ann Arbor, MI, United States; ^2^ Department of Computational Medicine and Bioinformatics, University of Michigan, Ann Arbor, MI, United States; ^3^ Department of Biology and Center for Life in Extreme Environments, Portland State University, Portland, OR, United States; ^4^ Department of Biology, University of Texas at San Antonio, San Antonio, TX, United States

**Keywords:** asRNA, anti-sense RNA, pathogenesis, virulence, bacteria, regulation, riboregulation

## Abstract

Regulatory RNAs contribute to gene expression control in bacteria. Antisense RNAs (asRNA) are a class of regulatory RNAs that are transcribed from opposite strands of their target genes. Typically, these untranslated transcripts bind to cognate mRNAs and rapidly regulate gene expression at the post-transcriptional level. In this article, we review asRNAs that modulate bacterial fitness and increase virulence. We chose examples that underscore the variety observed in nature including, plasmid- and chromosome-encoded asRNAs, a riboswitch-regulated asRNA, and asRNAs that require other RNAs or RNA-binding proteins for stability and activity. We explore how asRNAs improve bacterial fitness and virulence by modulating plasmid acquisition and maintenance, regulating transposon mobility, increasing resistance against bacteriophages, controlling flagellar production, and regulating nutrient acquisition. We conclude with a brief discussion on how this knowledge is helping to inform current efforts to develop new therapeutics.

## Introduction

A major breakthrough in biology was the discovery of non-coding RNAs (ncRNAs) that regulate gene expression instead of coding for proteins. ncRNAs play important regulatory roles in all domains of life (Katayama et al., 2005; Beiter et al., 2009; Georg and Hess, 2011; Lybecker et al., 2014). In bacteria, ncRNAs regulate gene expression at the post-transcriptional level by binding to messenger RNAs (mRNAs) to control several processes, including pathogenesis (Gripenland et al., 2010; Gottesman and Storz, 2011; Kacharia et al., 2017). Typically, ncRNAs that are encoded on the opposite strands of target genes (complementary to sense transcript) are known as *cis*-acting antisense RNAs (asRNAs), while an ncRNA that is encoded in a separate part of the genome in relation to its target mRNAs is called a *trans*-acting small RNA (sRNA) (Waters and Storz, 2009; Thomason and Storz, 2010; Georg and Hess, 2011). Regulatory RNAs generally have an advantage over regulatory proteins because their synthesis require lower energy and they act rapidly. In addition, their co-degradation along with target mRNAs allow precise control of regulatory circuits, which is key for bacteria to quickly adapt to host immune response (Storz et al., 2011; Updegrove et al., 2015). asRNAs are particularly useful for rapid gene regulation because they bind to target mRNAs with perfect complementarity, whereas sRNAs form imperfect complementarity with target mRNAs and often require chaperone proteins such as Hfq and ProQ for stability and function (Georg and Hess, 2011; Saberi et al., 2016; Hoynes-O’Connor and Moon, 2016; Dutcher and Raghavan, 2018).

Initially, asRNAs were thought to be rare in bacteria, and the pervasive antisense transcription observed in microarray-based studies were assumed to be experimental artifacts (Johnson et al., 2005; Perocchi et al., 2007; Georg and Hess, 2011). Even with the advent of high-throughput sequencing, it was initially difficult to differentiate between bona fide asRNAs and transcriptional noise because of low sequence coverage (Georg et al., 2009; Yamaguchi et al., 2011; Georg and Hess, 2018). With the increase in sequencing resolution, recent studies have confirmed the presence of abundant asRNAs in bacteria and have revealed it to be a genome-wide phenomenon (Dornenburg et al., 2010; Georg and Hess, 2011; Raghavan et al., 2012; Thomason et al., 2015).

asRNAs have been shown to modulate bacterial pathogenicity by either regulating the expression of virulence genes (Giangrossi et al., 2010) or by controlling biochemical processes that improve bacterial fitness, which in turn boosts virulence (Gripenland et al., 2010; Lejars et al., 2019). In this mini review, we focus on the latter. In particular, we cover examples where we generally understand the mechanism of action and where the genome locations of asRNAs have been determined. We also chose examples that underscore the variety observed in nature, including short asRNAs and long asRNAs, those found in plasmids and those encoded on genomes, asRNAs that require binding stability from other RNAs or proteins, and asRNAs that work in concert with riboswitches. These examples are presented in several sections based on the main roles asRNAs play in pathogenesis: 1) acquisition and regulation of virulence plasmids, 2) modulation of transposon mobility, 3) increasing resistance against bacteriophages, 4) controlling flagellar production, and 5) regulating nutrient acquisition.

## Modes of Antisense RNA-Based Improvement in Fitness and Virulence

### Acquisition and Maintenance of Virulence Plasmids

**Figure 1:**
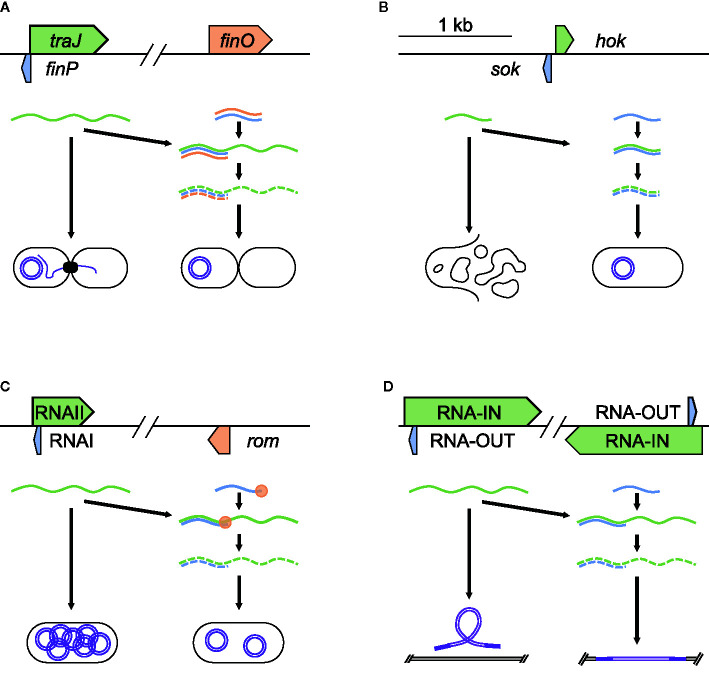
Acquisition and regulation of virulence plasmids and modulation of transposons. Examples of asRNA post-transcriptional regulation of acquisition and regulation of virulence plasmids and modulation of transposons. **(A)** *finP* control of plasmid acquisition through conjugation in *E. coli* F plasmid (NC_002483.1), **(B)** *hok*/*sok* toxin-antitoxin to maintain plasmids in *S. flexneri* R100 plasmid (NC_002134.1), **(C)** RNAI control of plasmid copy number in *E. coli* pColK plasmid (NC_006881.1), and **(D)** RNA-OUT regulation of transposase expression, controlling transposon movement in *S. flexneri* R100 plasmid (NC_002134.1). Each panel shows the relative position and size of the asRNA (blue), the target it regulates (green), and any factors required for stable binding (orange). In these examples, the successful binding of asRNA to its target promotes degradation.

A major avenue through which bacteria acquire new virulence factors is by obtaining new plasmids *via* conjugation, a process that involves asRNAs. An example of this is F-like plasmids, which are part of a large group of conjugative plasmids frequently found in *Escherichia coli* and throughout Enterobacteriaceae (Jerome et al., 1999). These plasmids frequently harbor accessory genes, including antibiotic resistance genes, enterotoxins, and other virulence genes (Koraimann, 2018). Conjugation is encoded by the Tra-operon, with initiation requiring TraJ. Initiation is regulated by FinOP, which consists of the RNA binding protein FinO and FinP, an asRNA. FinP attaches to the ribosome binding site of *traJ*, inhibiting its translation and promoting mRNA degradation (**Figure 1A**). FinO contributes by helping promote FinP binding to *traJ*, as well as protecting FinP from RNase E cleavage (Arthur et al., 2003). FinP levels are controlled by RNase E digestion, preventing binding to *traJ*. These processes play out temporally during conjugation, starting with initial high level of *traJ* expression, followed by dampening and repression, maintaining bacterial fitness by reducing the metabolic burden of the plasmid (Glover et al., 2015).

Once bacteria acquire advantageous virulence factors through plasmids, some plasmids are retained through toxin/antitoxin systems (Gerdes and Wagner, 2007). These systems function by encoding a toxin and a paired strong antitoxin — many of which function as asRNAs, on the plasmid. During cell division, loss of the plasmid in a daughter cell results in loss of the strong antitoxin, leading to the death of cells without a plasmid copy. A well-studied system is the *hok*/*sok* system of R1 plasmids in *E. coli* and R100 in *Shigella flexneri*, known for harboring various antibiotic resistance genes (Ogata and Levine, 1980; Cox and Schildbach, 2017). This system encodes the Hok (host killing) toxin, which leads to cell death by depolarization of the cell membrane (Pecota et al., 2003), and Sok (suppression of killing), an asRNA antitoxin, which degrades very quickly (Gerdes and Wagner, 2007). Sok acts by binding to *hok* mRNA to block translation of the toxin (**Figure 1B**). Within the *E. coli* chromosome, *sok* gene has a very weak promoter, resulting in the production of small amounts of antitoxin that are degraded quickly and are unable to keep up with the Hok toxin, leading to cell death. On the R1 plasmid, the *sok* gene has a strong promoter, producing many times more of Sok than Hok (Gerdes et al., 1990). Hence, if the cell contains an R1 plasmid, excess Sok continues to bind all of Hok and prevent cell death. This ensures that after cell division, *E. coli* daughter cells will survive only if they maintain the plasmid. Thus, the Sok asRNA maintains bacterial fitness by promoting the retention of the R1/R100 plasmid, which has been found to improve bacterial stress response and growth in growth-limiting conditions (Chukwudi and Good, 2015).

Replication control is another asRNA-based mechanism used by bacteria to maintain plasmids. The presence of too many copies of a plasmid can increase the metabolic burden of the cell, lowering fitness through reduced growth rate and weakened competitiveness (Baltrus, 2013; Vogwill and MacLean, 2015). However, too few plasmid copies could result in the loss of a potentially useful plasmid in subsequent generations (Millan and MacLean, 2017; Pluta and Espinosa, 2018). Bacteria encode plasmid copy number control systems in order to maintain optimal number of plasmids. One that has been widely studied is found in ColE1-related plasmids (Lacatena and Cesareni, 1981) present in *E. coli*. The plasmid is named for containing the gene that encodes Colicin E1, the product of which is active against *E. coli*, as well as containing a gene for conferring immunity to Colicin E1. Under stressful conditions such as nutrient depletion, overcrowding, or antibiotics *E. coli* express Colicin E1, which promotes bacterial proliferation in mixed microbe niches such as the intestinal tract (Spangler et al., 1985; Riley and Gordon, 1999). To replicate the plasmid, RNAII (a pre-primer) attaches to DNA at origin of replication. RNAII is then trimmed into a primer, which initializes plasmid replication. The 5′ region of RNAII contains the asRNA RNAI, which inhibits ColE1 plasmid replication (**Figure 1C**). RNAI inhibits plasmid replication with the help of the Rom protein by binding to RNAII, preventing RNAII from binding to the plasmid origin of replication. As the copy number of ColE1 plasmid increases, so does the concentration of RNAI, resulting in a balance of copy number through negative control (del Solar and Espinosa, 2000). This ensures that there are enough copies of the virulence plasmid to pass on to daughter cells, while maintaining fitness by reducing the metabolic burden of what are often large — sometimes hundreds of kilobases long, plasmids (Sengupta and Austin, 2011).

### Regulation of Transposon Mobility

Another role for asRNAs in maintaining bacterial fitness and virulence is by controlling the movement of transposons, which are genetic elements that move from one position to another within a genome (Bourque et al., 2018). Insertion of transposons in virulence associated genes could reduce a bacterium’s pathogenicity or increase its susceptibility to antibiotics (Murray et al., 2009; Murray et al., 2015; Kalindamar et al., 2019). In addition, transposable elements could modulate virulence by affecting biofilm formation (Arciola et al., 2004; Kiem et al., 2004; Perez et al., 2015) and reduce fitness by interrupting metabolic genes (Lapierre et al., 2002; Christie-Oleza et al., 2008; Moffatt et al., 2011). Bacteria defend against this by controlling transposases, the enzymes required for transposons’ mobility. An example of inhibition of bacterial transposase can be seen in the Tn10 transposable element, which is found in *S. flexneri* and other Enterobacteriaceae (Ma and Simons, 1990). Tn10 contains a number of tetracycline resistance genes and a pair of IS10 insertion sequences that each encode transposases that promote transposon mobility. IS10s also encode antisense RNA (RNA-OUT), which is found in the 5′-most segment of the transposase mRNA (RNA‐IN). RNA-OUT inhibits transposase translation by binding RNA‐IN and blocking ribosome binding site (**Figure 1D**). As the Tn10 copy number increases, asRNA increases to suppress the transposition of the transposon (Ellis et al., 2015); thus, bacteria are able to maintain fitness by reducing the chance of mobile elements disrupting essential genes or virulence factors.

### Modulation of Bacteriophages

**Figure 2:**
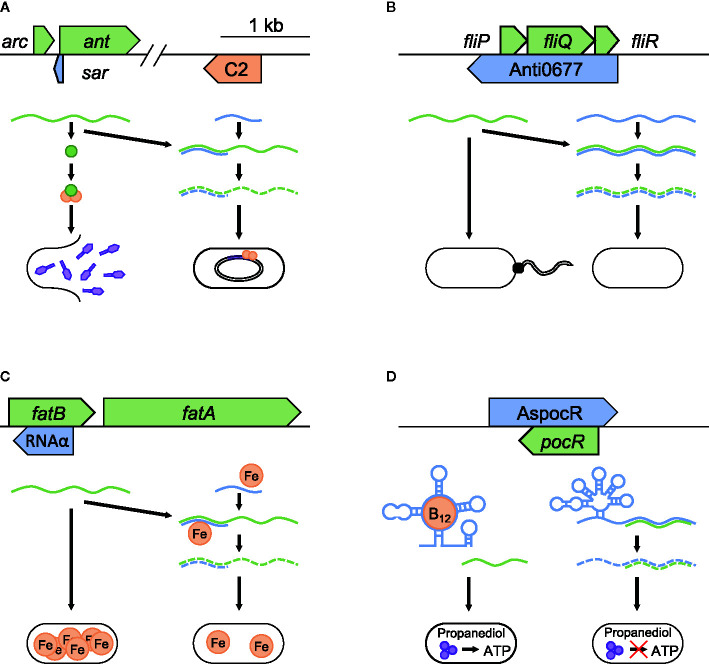
Modulation of bacteriophages, flagella production, and nutrient acquisition. Examples of asRNA post-transcriptional regulation in pathogenic bacteria to increase fitness during infection. Repression of temperate bacteriophages is seen in **(A)** *sar* repression of lytic growth in Enterobacteria P22 phage (NC_002371.2). *sar* blocks the anti-repressor *ant* from binding to C2 (orange), which prevents escape from lysogenic growth. Temperature dependent regulation in bacteria is represented by **(B)** Anti0677 control of flagella formation in response to temperature change in *L. monocytogenes* (NC_003210.1). Examples that highlight regulation of nutrient acquisition can be seen in **(C)** RNAα control of iron acquisition in *V. anguillarum* pJM1 plasmid (NC_005250.1) and **(D)** AspocR/B_12_ riboswitch control of opportunistic propanediol catabolism in *L. monocytogenes* (NC_003210.1). Each panel shows the relative position and size of the asRNA (blue), and the target it regulates (green). In **(A–C)**, the successful binding of the asRNA to its target promotes mRNA degradation, with iron required in **(C)** for stable binding. In **(D)**, when B_12_ is absent, a full-length version of the asRNA is transcribed, which binds to pocR mRNA and blocks propanediol fermentation. When B_12_ is present and binds to the riboswitch, transcription ends prematurely, resulting in the production of PocR, which promotes propanediol fermentation.

During infection, pathogenic bacteria have to outcompete other bacteria that share their niche and defend against both internal and external threats. One of the major dangers that bacteria face is from bacteriophages, many of which have lysogenic and lytic growth cycles (Echols, 1972). In the lysogenic cycle, phage DNA is integrated into the bacterial chromosome, allowing replication of the phage to occur more passively along with that of the bacterium. In lytic reproduction, the phage actively creates a large number of progeny and quickly lyse the bacterial cell to continue its lifecycle. Temperate phages include both cycles and are found in about half of microbial genomes currently sequenced (Touchon et al., 2016). While attempting to block infection by phages with lytic growth cycles often reduces virulence and fitness, allowing temperate phages to stay in the lysogenic cycle benefit bacteria by delaying eventual cell lysis (Seed et al., 2014; León and Bastías, 2015; Harrison and Brockhurst, 2017; Howard-Varona et al., 2017). An example of this can be seen in *Salmonella*’s maintenance of lysogeny in P22 phages (Liao et al., 1987). In P22, lytic growth is inhibited by the regulatory protein C2, which blocks the transcription of proteins needed for the development of lytic cycle. The switch to lytic growth is brought on by the anti-repressor protein Ant, which blocks C2 binding to the O_R_ and O_L_ operators of the P22 phage. Repression of this progression into the lytic replication cycle can be accomplished through Sar, an asRNA in the intergenic region of *arc*-*ant* mRNA (Schaefer and McClure, 1997). Sar blocks the *ant* ribosome binding site, which results in a failure to produce Ant and thus increasing bacterial fitness by preventing the escape of the prophage from the lysogenic state (**Figure 2A**).

### Control of Flagella Production

By modifying their outer structures bacteria evade immune response and improve persistence within hosts. An example of asRNA’s involvement in this process is observed in *Listeria monocytogenes*, where a long asRNA regulates flagella production in response to temperature (Toledo-Arana et al., 2009). At 30°C, *L. monocytogenes* expresses flagella on its surface and exhibits swimming motility. Producing flagella requires the expression of a number of genes, including the flagellum export apparatus genes (*fliP*, *fliQ*, and *fliR*). When the temperature rises to 37°C, the motility gene repressor *mogR* switches off flagella formation (Lebreton and Cossart, 2017). Overlapping *fliP*, *fliQ*, and *fliR* is a large asRNA, Anti0677, which negatively regulates their expression by promoting mRNA degradation by direct interaction (**Figure 2B**). Additionally, the end of Anti0677 both contains the coding sequence for and drives the expression of MogR. These two effects — the antisense component Anti0677 and increased expression of MogR — together suppress flagella formation within the host, possibly reducing the host inflammatory response attempting to lyse the invading bacteria (Hayashi et al., 2001). The term “excludon” has been proposed for transcripts such as Anti0677 that both code for proteins and regulate the expression of multiple genes or operons encoded divergently from them (Sesto et al., 2013).

### Regulation of Nutrient Acquisition

Bacteria can optimize their growth rates by modulating nutrient acquisition. This capability bestows increased fitness to pathogens by allowing them to survive under nutrient-poor conditions such as infections (Parrow et al., 2013; Fonseca and Swanson, 2014). An example of this phenomenon is iron uptake suppression in the fish pathogen *Vibrio anguillarum* (Chen and Crosa, 1996; Sesto et al., 2013). Iron is an essential nutrient for most bacteria because it plays critical roles in numerous metabolic processes (León-Sicairos et al., 2015). *V. anguillarum* contains the pJM1 plasmid, which encodes most genes necessary for iron-anguibactin siderophore transport and biosynthesis (Naka et al., 2010). Among these are transport proteins FatA and FatB, which are encoded by genes *fatA* and *fatB* that are transcribed together as a polycistronic mRNA. An asRNA termed RNAα, which is encoded within *fatB*, is expressed in response to increasing iron levels. It binds to the *fatB* portion of the *fatA*-*fatB* mRNA and represses the translation of both genes (**Figure 2C**). Iron further stabilizes the binding of RNAα to *fatA*-*fatB* mRNA, leading to its degradation. This system helps to reduce the fitness cost associated with metabolic burden by synthesizing iron siderophores only when confronted with iron-poor conditions, thereby allowing the bacterium to optimize its resources to outcompete other bacteria.

Another example of improving metabolic fitness through regulating bacterial nutrient acquisition is the regulation of propanediol catabolism in *L. monocytogenes* (Mellin et al., 2013). Propanediol is a byproduct of the fermentation of rhamnose and fucose, and is often produced by commensal bacteria in host intestines (Bobik et al., 1997; Degnan et al., 2014). Propanediol fermentation is facilitated through a coenzyme B_12_-dependent process and can support bacterial growth by providing ATP (Toraya et al., 1979). Some studies suggest that propanediol catabolism gives bacteria a competitive advantage, with mutations in related genes resulting in a virulence defect (Conner et al., 1998). Within *L. monocytogenes*, the presence of propanediol activates the transcription factor PocR, which controls the expression of propanediol catabolism genes that require vitamin B_12_ as a cofactor. On the opposite strand of *pocR* gene, there is a vitamin B_12_ riboswitch-regulated asRNA, AspocR (**Figure 2D**). When vitamin B_12_ is absent, a full-length version of AspocR is transcribed, which inhibits *pocR* expression. When vitamin B_12_ is bound to the riboswitch, AspocR transcript ends prematurely, and hence cannot inhibit *pocR*. This leads to the production of PocR, which promotes the expression of propanediol catabolism genes. Thus, the riboswitch-regulated asRNA allows the expression of propanediol fermentation genes only when both propanediol and B_12_ are present, thereby reducing the fitness cost associated with unnecessary metabolic burden.

## Conclusions

asRNAs are ubiquitous in bacteria and are involved in a multitude of pathogenesis-related mechanisms. The wide range of asRNA functions span the control of intra- and extra-chromosomal DNA, as well as adaption strategies to improve persistence under changing environments. Some asRNAs are only found in specific bacterial species, while others are found across bacteria. Because asRNAs play important roles in modulating the fitness of pathogenic bacteria, current research is focused not only on identifying new asRNAs, but also to use them to our advantage by developing novel asRNA-based therapeutics. For instance, bacterial antibiotic resistance genes can be targeted with synthetic asRNAs, resulting in antibiotic sensitive bacteria (Ji et al., 2004; Nikravesh et al., 2007). Other possible applications include using asRNA to silence bacterial metabolism or ribosomal protein coding genes (successfully shown in *E. coli*) and protection from bacteriophages in the production of live mucosal vaccines (Sturino and Klaenhammer, 2006; Alessandra et al., 2008; Suzukia et al., 2020). Applications of these techniques beyond *in vitro* studies have been limited due to difficulties in delivering asRNAs to the site of infection (Good and Stach, 2011; Saberi et al., 2016). As these impediments are addressed, the use of asRNAs in therapeutics will likely expand and contribute to the understanding of the rich landscape of bacterial control systems.

## Author Contributions

JM drafted the initial manuscript and figures. JM and RR read and edited the initial manuscript. All authors contributed to the article and approved the submitted version.

## Funding

JM is supported by a grant from the National Science Foundation GRFP (DGE‐1256260) and through the Rackham Merit Fellowship through the University of Michigan. RR is supported by NIH grants AI133023 and DE028409.

## Conflict of Interest

The authors declare that the research was conducted in the absence of any commercial or financial relationships that could be construed as a potential conflict of interest.

## References

[B1] Alessandra S. Alessandro T. Flavio S. Alejandro H. (2008). Artificial antisense RNAs silence *lacZ* in *E. coli* by decreasing target mRNA concentration. BMB Rep. 41, 568–574. 10.5483/bmbrep.2008.41.8.568 18755071

[B2] Arciola C. R. Campoccia D. Gamberini S. Rizzi S. Donati M. E. Baldassarri L.. (2004). Search for the insertion element IS256 within the *ica* locus of *Staphylococcus epidermidis* clinical isolates collected from biomaterial-associated infections. Biomaterials 25, 4117–4125. 10.1016/j.biomaterials.2003.11.027 15046902

[B3] Arthur D. C. Ghetu A. F. Gubbins M. J. Edwards R. A. Frost L. S. Glover J. N. (2003). FinO is an RNA chaperone that facilitates sense-antisense RNA interactions. EMBO J. 22, 6346–6355. 10.1093/emboj/cdg607 14633993 PMC291848

[B4] Baltrus D. A. (2013). Exploring the costs of horizontal gene transfer. Trends Ecol. Evol. 28, 489–495. 10.1016/j.tree.2013.04.002 23706556

[B5] Beiter T. Reich E. Williams R. W. Simon P. (2009). Antisense transcription: A critical look in both directions. Cell Mol. Life Sci. 66, 94–112. 10.1007/s00018-008-8381-y 18791843 PMC11131530

[B6] Bobik T. A. Xu Y. Jeter R. M. Otto K. E. Roth J. R. (1997). Propanediol utilization genes (pdu) of *Salmonella typhimurium*: Three genes for the propanediol dehydratase. J. Bacteriol. 179, 6633–6639. 10.1128/jb.179.21.6633-6639.1997 9352910 PMC179589

[B7] Bourque G. Burns K. H. Gehring M. Gorbunova V. Seluanov A. Hammell M.. (2018). Ten things you should know about transposable elements. Genome Biol. 19, 199. 10.1186/s13059-018-1577-z 30454069 PMC6240941

[B8] Chen Q. Crosa J. H. (1996). Antisense RNA, fur, iron, and the regulation of iron transport genes in *Vibrio anguillarum* . J. Biol. Chem. 271, 18885–18891. 10.1074/jbc.271.31.18885 8702549

[B9] Christie-Oleza J. A. Nogales B. Martín-Cardona C. Lanfranconi M. P. Albertí S. Lalucat J.. (2008). IS*Pst*9, an ISL3-like insertion sequence from *Pseudomonas stutzeri* AN10 involved in catabolic gene inactivation. Int. Microbiol. 11, 101–110. 10.2436/20.1501.01.49 18645960

[B10] Chukwudi C. U. Good L. (2015). The role of the *hok/sok* locus in bacterial response to stressful growth conditions. Microb. Pathog. 79, 70–79. 10.1016/j.micpath.2015.01.009 25625568

[B11] Conner C. P. Heithoff D. M. Julio S. M. Sinsheimer R. L. Mahan M. J. (1998). Differential patterns of acquired virulence genes distinguish *Salmonella* strains. Proc. Natl. Acad. Sci. 95, 4641–4645. 10.1073/pnas.95.8.4641 9539791 PMC22543

[B12] Cox K. E. L. Schildbach J. F. (2017). Sequence of the R1 plasmid and comparison to F and R100. Plasmid 91, 53–60. 10.1016/j.plasmid.2017.03.007 28359666

[B13] Degnan P. H. Taga M. E. Goodman A. L. (2014). Vitamin B_12_ as a modulator of gut microbial ecology. Cell Metab. 20, 769–778. 10.1016/j.cmet.2014.10.002 25440056 PMC4260394

[B14] del Solar G. Espinosa M. (2000). Plasmid copy number control: An ever-growing story. Mol. Microbiol. 37, 492–500. 10.1046/j.1365-2958.2000.02005.x 10931343

[B15] Dornenburg J. DeVita A. Palumbo M. Wade J. (2010). Widespread antisense transcription in *Escherichia coli* . MBio 1, e00024–e00010. 10.1128/mBio.00024-10 20689751 PMC2912661

[B16] Dutcher H. A. Raghavan R. (2018). Origin, evolution, and loss of bacterial small RNAs. Microbiol. Spectr. 6, RWR–0004-2017. 10.1128/microbiolspec.RWR-0004-2017 PMC5890949 29623872

[B17] Echols H. (1972). Developmental pathways for the temperate phage: Lysis vs lysogeny. Annu. Rev. Genet. 6, 157–190. 10.1146/annurev.ge.06.120172.001105 4604314

[B18] Ellis M. J. Trussler R. S. Haniford D. B. (2015). Hfq binds directly to the ribosome-binding site of IS10 transposase mRNA to inhibit translation. Mol. Microbiol. 96, 633–650. 10.1111/mmi.12961 25649688 PMC5006887

[B19] Fonseca M. V. Swanson M. S. (2014). Nutrient salvaging and metabolism by the intracellular pathogen *Legionella pneumophila* . Front. Cell. Infect. Microbiol. 4:12. 10.3389/fcimb.2014.00012 24575391 PMC3920079

[B20] Georg J. Hess W. R. (2011). *cis*-antisense RNA, another level of gene regulation in bacteria. Microbiol. Mol. Biol. Rev. 75, 286–300. 10.1128/MMBR.00032-10 21646430 PMC3122628

[B21] Georg J. Hess W. R. (2018). Widespread antisense transcription in prokaryotes. Microbiol. Spectr. 6, RWR–0029. 10.1128/microbiolspec.RWR-0029-2018 PMC11633618 30003872

[B22] Georg J. Voss B. Scholz I. Mitschke J. Wilde A. Hess W. R. (2009). Evidence for a major role of antisense RNAs in cyanobacterial gene regulation. Mol. Syst. Biol. 5, 305. 10.1038/msb.2009.63 19756044 PMC2758717

[B23] Gerdes K. Wagner E. G. (2007). RNA antitoxins. Curr. Opin. Microbiol. 10, 117–124. 10.1016/j.mib.2007.03.003 17376733

[B24] Gerdes K. Thisted T. Martinussen J. (1990). Mechanism of post-segregational killing by the *hok*/*sok* system of plasmid R1: *sok* antisense RNA regulates formation of a *hok* mRNA species correlated with killing of plasmid-free cells. Mol. Microbiol. 4, 1807–1818. 10.1111/j.1365-2958.1990.tb02029.x 1707122

[B25] Giangrossi M. Prosseda G. Tran C. N. Brandi A. Colonna B. Falconi M. (2010). A novel antisense RNA regulates at transcriptional level the virulence gene *icsA of Shigella flexneri* . Nucleic. Acids Res. 38, 3362–3375. 10.1093/nar/gkq025 20129941 PMC2879508

[B26] Glover J. N. Chaulk S. G. Edwards R. A. Arthur D. Lu J. Frost L. S. (2015). The FinO family of bacterial RNA chaperones. Plasmid 78, 79–87. 10.1016/j.plasmid.2014.07.003 25102058

[B27] Good L. Stach J. E. (2011). Synthetic RNA silencing in bacteria - antimicrobial discovery and resistance breaking. Front. Microbiol. 2, 185. 10.3389/fmicb.2011.00185 21941522 PMC3170882

[B28] Gottesman S. Storz G. (2011). Bacterial small RNA regulators: Versatile roles and rapidly evolving variations. Cold Spring Harb. Perspect. Biol. 3, a003798. 10.1101/cshperspect.a003798 20980440 PMC3225950

[B29] Gripenland J. Netterling S. Loh E. Tiensuu T. Toledo-Arana A. Johansson J. (2010). RNAs: Regulators of bacterial virulence. Nat. Rev. Microbiol. 8, 857–866. 10.1038/nrmicro2457 21079634

[B30] Harrison E. Brockhurst M. A. (2017). Ecological and evolutionary benefits of temperate phage: What does or doesn’t kill you makes you stronger. Bioessays 39:1700112. 10.1002/bies.201700112 28983932

[B31] Hayashi F. Smith K. D. Ozinsky A. Hawn T. R. Yi E. C. Goodlett D. R.. (2001). The innate immune response to bacterial flagellin is mediated by Toll-like receptor 5. Nature 410, 1099–1103. 10.1038/35074106 11323673

[B32] Howard-Varona C. Hargreaves K. R. Abedon S. T. Sullivan M. B. (2017). Lysogeny in nature: Mechanisms, impact and ecology of temperate phages. ISME J. 11, 1511–1520. 10.1038/ismej.2017.16 28291233 PMC5520141

[B33] Hoynes-O’Connor A. Moon T. S. (2016). Development of design rules for reliable antisense RNA behavior in *E. coli* . ACS Synth. Biol. 5, 1441–1454. 10.1021/acssynbio.6b00036 27434774

[B34] Jerome L. J. van Biesen T. Frost L. S. (1999). Degradation of FinP antisense RNA from F-like plasmids: The RNA-binding protein, FinO, protects FinP from ribonuclease E. J. Mol. Biol. 285, 1457–1473. 10.1006/jmbi.1998.2404 9917389

[B35] Ji Y. Yin D. Fox B. Holmes D. J. Payne D. Rosenberg M. (2004). Validation of antibacterial mechanism of action using regulated antisense RNA expression in *Staphylococcus aureus* . FEMS Microbiol. Lett. 231, 177–184. 10.1016/S0378-1097(03)00931-5 14987762

[B36] Johnson J. M. Edwards S. Shoemaker D. Schadt E. E. (2005). Dark matter in the genome: Evidence of widespread transcription detected by microarray tiling experiments. Trends Genet. 21, 93–102. 10.1016/j.tig.2004.12.009 15661355

[B37] Kacharia F. R. Millar J. A. Raghavan R. (2017). Emergence of new sRNAs in enteric bacteria is associated with low expression and rapid evolution. J. Mol. Evol. 84, 204–213. 10.1007/s00239-017-9793-9 28405712

[B38] Kalindamar S. Lu J. Abdelhamed H. Tekedar H. C. Lawrence M. L. Karsi A. (2019). Transposon mutagenesis and identification of mutated genes in growth-delayed *Edwardsiella ictaluri* . BMC Microbiol. 19, 55. 10.1186/s12866-019-1429-3 30849940 PMC6408766

[B39] Katayama S. Tomaru Y. Kasukawa T. Waki K. Nakanishi M. Nakamura M.. (2005). Antisense transcription in the mammalian transcriptome. Science 309, 1564–1566. 10.1126/science.1112009 16141073

[B40] Kiem S. Oh W. S. Peck K. R. Lee N. Y. Lee J. Song J.. (2004). Phase variation of biofilm formation in *Staphylococcus aureus* by IS*256* insertion and its impact on the capacity adhering to polyurethane surface. J. Korean Med. Sci. 19, 779–782. 10.3346/jkms.2004.19.6.779 15608385 PMC2816298

[B41] Koraimann G. (2018). Spread and persistence of virulence and antibiotic resistance genes: A ride on the F plasmid conjugation module. EcoSal Plus 8. 10.1128/ecosalplus.ESP-0003-2018 PMC11575672 30022749

[B42] Lacatena R. M. Cesareni G. (1981). Base pairing of RNA I with its complementary sequence in the primer precursor inhibits ColE1 replication. Nature 294, 623–626. 10.1038/294623a0 6171736

[B43] Lapierre L. Mollet B. Germond J. (2002). Regulation and adaptive evolution of lactose operon expression in *Lactobacillus delbrueckii* . J. Bacteriol. 184, 928–935. 10.1128/jb.184.4.928-935.2002 11807052 PMC134810

[B44] Lebreton A. Cossart P. (2017). RNA- and protein-mediated control of *Listeria monocytogenes* virulence gene expression. RNA Biol. 14, 460–470. 10.1080/15476286.2016.1189069 27217337 PMC5449094

[B45] Lejars M. Kobayashi A. Hajnsdorf E. (2019). Physiological roles of antisense RNAs in prokaryotes. Biochimie 164, 3–16. 10.1016/j.biochi.2019.04.015 30995539

[B46] León M. Bastías R. (2015). Virulence reduction in bacteriophage resistant bacteria. Front. Microbiol. 6, 343. 10.3389/fmicb.2015.00343 25954266 PMC4407575

[B47] León-Sicairos N. Angulo-Zamudio U. A. de la Garza M. Velázquez-Román J. Flores-Villaseñor H. M. Canizalez-Román A. (2015). Strategies of *Vibrio parahaemolyticus* to acquire nutritional iron during host colonization. Front. Microbiol. 6:702. 10.3389/fmicb.2015.00702 26217331 PMC4496571

[B48] Liao S. M. Wu T. H. Chiang C. H. Susskind M. M. McClure W. R. (1987). Control of gene expression in bacteriophage P22 by a small antisense RNA. I. Characterization in vitro of the Psar promoter and the sar RNA transcript. Genes Dev. 1, 197–203. 10.1101/gad.1.2.197 2445626

[B49] Lybecker M. Bilusic I. Raghavan R. (2014). Pervasive transcription: Detecting functional RNAs in bacteria. Transcription 5, e944039. 10.4161/21541272.2014.944039 25483405 PMC4581347

[B50] Ma C. Simons R. W. (1990). The IS10 antisense RNA blocks ribosome binding at the transposase translation initiation site. EMBO J. 9, 1267–1274. 10.1002/j.1460-2075.1990.tb08235.x 1691097 PMC551804

[B51] Mellin J. R. Tiensuu T. Bécavin C. Gouin E. Johansson J. Cossart P. (2013). A riboswitch-regulated antisense RNA in *Listeria monocytogenes* . Proc. Natl. Acad. Sci. 110, 13132–13137. 10.1073/pnas.1304795110 23878253 PMC3740843

[B52] Millan A. S. MacLean R. C. (2017). Fitness costs of plasmids: A limit to plasmid transmission. Microbiol. Spectr. 5, MTBP–0016. 10.1128/microbiolspec.MTBP-0016-2017 PMC11687550 28944751

[B53] Moffatt J. H. Harper M. Adler B. Nation R. L. Li J. Boyce J. D. (2011). Insertion sequence IS*Aba11* is involved in colistin resistance and loss of lipopolysaccharide in *Acinetobacter baumannii.* Antimicrob. Agents Chemother. 55, 3022–3024. 10.1128/AAC.01732-10 PMC3101452 21402838

[B54] Murray G. L. Morel V. Cerqueira G. M. Croda J. Srikram A. Henry R.. (2009). Genome-wide transposon mutagenesis in pathogenic *Leptospira species* . Infect. Immun. 77, 810–816. 10.1128/IAI.01293-08 19047402 PMC2632054

[B55] Murray J. L. Kwon T. Marcotte E. M. Whiteley M. (2015). Intrinsic antimicrobial resistance determinants in the superbug *Pseudomonas aeruginosa* . mBio 6, e01603–e01615. 10.1128/mBio.01603-15 26507235 PMC4626858

[B56] Naka H. López C. S. Crosa J. H. (2010). Role of the pJM1 plasmid-encoded transport proteins FatB, C and D in ferric anguibactin uptake in the fish pathogen *Vibrio anguillarum* . Environ. Microbiol. Rep. 2, 104–111. 10.1111/j.1758-2229.2009.00110.x 21304833 PMC3034151

[B57] Nikravesh A. Dryselius R. Faridani O. R. Goh S. Sadeghizadeh M. Behmanesh M.. (2007). Antisense PNA accumulates in *Escherichia coli* and mediates a long post-antibiotic effect. Mol. Ther. 15, 1537–1542. 10.1038/sj.mt.6300209 17534267

[B58] Ogata R. T. Levine R. R. (1980). Characterization of complement resistance in *Escherichia coli* conferred by the antibiotic resistance plasmid R100. J. Immunol. 125, 1494–1498. 6997381

[B59] Parrow N. L. Fleming R. E. Minnick M. F. (2013). Sequestration and scavenging of iron in infection. Infect. Immun. 81, 3503–3514. 10.1128/IAI.00602-13 23836822 PMC3811770

[B60] Pecota D. C. Osapay G. Selsted M. E. Wood T. K. (2003). Antimicrobial properties of the *Escherichia coli* R1 plasmid host killing peptide. J. Biotechnol. 100, 1–12. 10.1016/s0168-1656(02)00240-7 12413781

[B61] Perez M. Calles-Enríquez M. del Rio B. Ladero V. Martín M. C. Fernández M.. (2015). IS*256* abolishes gelatinase activity and biofilm formation in a mutant of the nosocomial pathogen *Enterococcus faecalis* V583. Can. J. Microbiol. 61, 517–519. 10.1139/cjm-2015-0090 25966618

[B62] Perocchi F. Xu Z. Clauder-Münster S. Steinmetz L. M. (2007). Antisense artifacts in transcriptome microarray experiments are resolved by actinomycin D. Nucleic Acids Res. 35, e128. 10.1093/nar/gkm683 17897965 PMC2095812

[B63] Pluta R. Espinosa M. (2018). Antisense and yet sensitive: Copy number control of rolling circle-replicating plasmids by small RNAs. Wiley Interdiscip. Rev. RNA 9, e1500. 10.1002/wrna.1500 30074293

[B64] Raghavan R. Sloan D. B. Ochman H. (2012). Antisense transcription is pervasive but rarely conserved in enteric bacteria. MBio 3, e00156–e00112. 10.1128/mBio.00156-12 22872780 PMC3419515

[B65] Riley M. A. Gordon D. M. (1999). The ecological role of bacteriocins in bacterial competition. Trends Microbiol. 7, 129–133. 10.1016/s0966-842x(99)01459-6 10203843

[B66] Saberi F. Kamali M. Najafi A. Yazdanparast A. Moghaddam M. M. (2016). Natural antisense RNAs as mRNA regulatory elements in bacteria: A review on function and applications. Cell Mol. Biol. Lett. 21, 6. 10.1186/s11658-016-0007-z 28536609 PMC5415839

[B67] Schaefer K. L. McClure W. R. (1997). Antisense RNA control of gene expression in bacteriophage P22. I. Structures of *sar* RNA and its target, *ant* mRNA. RNA 3, 141–156. 9042942 PMC1369469

[B68] Seed K. D. Yen M. Shapiro J. Hilaire I. J. Charles R. C. Teng J. E.. (2014). Evolutionary consequences of intra-patient phage predation on microbial populations. Elife 3, e03497. 10.7554/eLife.03497 25161196 PMC4141277

[B69] Sengupta M. Austin S. (2011). Prevalence and significance of plasmid maintenance functions in the virulence plasmids of pathogenic bacteria. Infect. Immun. 79, 2502–2509. 10.1128/IAI.00127-11 21555398 PMC3191983

[B70] Sesto N. Wurtzel O. Archambaud C. Sorek R. Cossart P. (2013). The excludon: A new concept in bacterial antisense RNA-mediated gene regulation. Nat. Rev. Microbiol. 11, 75–82. 10.1038/nrmicro2934 23268228

[B71] Spangler R. Zhang S. P. Krueger J. Zubay G. (1985). Colicin synthesis and cell death. J. Bacteriol. 163, 167–173. 10.1128/JB.163.1.167-173.1985 3891723 PMC219094

[B72] Storz G. Vogel J. Wassarman K. M. (2011). Regulation by small RNAs in bacteria: Expanding frontiers. Mol. Cell. 43, 880–891. 10.1016/j.pestbp.2011.02.012.Investigations 21925377 PMC3176440

[B73] Sturino J. M. Klaenhammer T. R. (2006). Engineered bacteriophage-defence systems in bioprocessing. Nat. Rev. Microbiol. 4, 395–404. 10.1038/nrmicro1393 16715051

[B74] Suzukia Y. Ishimoto T. Fujita S. Kiryu S. Wada M. Akatsuka T.. (2020). Antimicrobial antisense RNA delivery to F-pili producing multidrug-resistant bacteria via a genetically engineered bacteriophage. Biochem. Biophys. Res. Commun. 530, 533–540. 10.1016/j.bbrc.2020.06.088 32739024

[B75] Thomason M. Storz G. (2010). Bacterial antisense RNAs: How many are there and what are they doing? Annu. Rev. Genet. 44, 167–188. 10.1146/annurev-genet-102209-163523 20707673 PMC3030471

[B76] Thomason M. K. Bischler T. Eisenbart S. K. Förstner K. U. Zhang A. Herbig A.. (2015). Global transcriptional start site mapping using differential RNA sequencing reveals novel antisense RNAs in *Escherichia coli* . J. Bacteriol. 197, 18–28. 10.1128/JB.02096-14 25266388 PMC4288677

[B77] Toledo-Arana A. Dussurget O. Nikitas G. Sesto N. Guet-Revillet H. Balestrino D.. (2009). The *Listeria* transcriptional landscape from saprophytism to virulence. Nature 459, 950–956. 10.1038/nature08080 19448609

[B78] Toraya T. Honda S. Fukui S. (1979). Fermentation of 1,2-propanediol with 1,2-ethanediol by some genera of Enterobacteriaceae, involving coenzyme B_12_-dependent diol dehydratase. J. Bacteriol. 139, 39–47. 10.1128/JB.139.1.39-47.1979 378959 PMC216824

[B79] Touchon M. Bernheim A. Rocha E. P. (2016). Genetic and life-history traits associated with the distribution of prophages in bacteria. ISME J. 10, 2744–2754. 10.1038/ismej.2016.47 27015004 PMC5113838

[B80] Updegrove T. B. Shabalina S. A. Storz G. (2015). How do base-pairing small RNAs evolve? FEMS Microbiol. Rev. 39, 379–391. 10.1093/femsre/fuv014 25934120 PMC4542690

[B81] Vogwill T. MacLean R. C. (2015). The genetic basis of the fitness costs of antimicrobial resistance: A meta-analysis approach. Evol. Appl. 8, 284–295. 10.1111/eva.12202 25861386 PMC4380922

[B82] Waters L. S. Storz G. (2009). Regulatory RNAs in bacteria. Cell 136, 615–628. 10.1016/j.cell.2009.01.043 19239884 PMC3132550

[B83] Yamaguchi Y. Park J. Inouye M. (2011). Toxin-antitoxin systems in bacteria and archaea. Annu. Rev. Genet. 45, 61–79. 10.1146/annurev-genet-110410-132412 22060041

